# A Cheminformatics Study Regarding the Human Health Risks Assessment of the Stereoisomers of Difenoconazole

**DOI:** 10.3390/molecules27154682

**Published:** 2022-07-22

**Authors:** Denisa Ioana Voiculescu, Diana Larisa Roman, Vasile Ostafe, Adriana Isvoran

**Affiliations:** 1 Department of Biology-Chemistry, Faculty of Chemistry, Biology, Geography, West University of Timisoara, 16 Pestalozzi, 300115 Timisoara, Romania; denisa.voiculescu94@e-uvt.ro (D.I.V.); diana.roman@e-uvt.ro (D.L.R.); vasile.ostafe@e-uvt.ro (V.O.); 2 Advanced Environmental Research Laboratories (AERL), 4 Oituz, 300086 Timisoara, Romania

**Keywords:** stereoisomers of difenoconazole, biological effects, toxicology, human health

## Abstract

Difenoconazole is a chemical entity containing two chiral centers and having four stereoisomers: (2*R*,4*R*)-, (2*R*,4*S*)-, (2*S*,4*R*)- and (2*S*,4*S*)-difenoconazole, the marketed product containing a mixture of these isomers. Residues of difenoconazole have been identified in many agricultural products and drinking water. A computational approach has been used to evaluate the toxicological effects of the difenoconazole stereoisomers on humans. It integrates predictions of absorption, distribution, metabolism, excretion and toxicity (ADMET) profiles, prediction of metabolism sites, and assessment of the interactions of the difenoconazole stereoisomers with human cytochromes, nuclear receptors and plasma proteins by molecular docking. Several toxicological effects have been identified for all the difenoconazole stereoisomers: high plasma protein binding, inhibition of cytochromes, possible hepatotoxicity, neurotoxicity, mutagenicity, skin sensitization potential, moderate potential to produce endocrine disrupting effects. There were small differences in the predicted probabilities of producing various biological effects between the distinct stereoisomers of difenoconazole. Furthermore, there were significant differences between the interacting energies of the difenoconazole stereoisomers with plasma proteins and human cytochromes, the spectra of the hydrogen bonds and aromatic donor–acceptor interactions being quite distinct. Some distinguishing results have been obtained for the (*2S*,*4S*)-difenoconazole: it registered the highest value for clearance, exposed reasonable probabilities to produce cardiotoxicity and carcinogenicity and negatively affected numerous nuclear receptors.

## 1. Introduction

Pesticides are chemicals largely used in agriculture for increasing crop production. Fungicides are a type of pesticide used worldwide in agriculture for preventive and curative purposes [1]. The excessive use of pesticides can lead to the accumulation of their residues in many agricultural products. Specific literature emphasizes evidence that fungicide residues are identified in fruits and/or vegetables [2,3,4,5]. Consequently, fungicides would exert human health risks in all situations of occupational, residential and environmental exposure and especially in the case of chronic exposure: negative effect on gut microbiota and metabolism disorders [6], neurotoxicity, carcinogenicity, reproductive and developmental toxicity [3,7,8].

This study focuses on difenoconazole, a commonly used triazole fungicide for grain crops, vegetables and fruits, and other crops [9]. Residues of the triazole fungicides were identified in numerous agricultural products [10] and were found to have adverse effects on both humans [11,12] and the environment [13]. A computational study exposed that several triazole fungicides showed good oral bioavailability, can penetrate the blood–brain barrier, impair the activity of hepatic cytochromes and reveal potential of cardiotoxicity, and skin sensitization and endocrine disruption [12]. Animal studies also emphasized that triazole fungicides may affect the endocrine system [11,14], and the central nervous system and produce hyperactivity [15].

Regarding difenoconazole, no negligible residues were identified in numerous fruits and vegetables, including grapes and sugar beets [9], watermelon (0.04–0.1 mg/kg) [16], apples [17], guava fruit (81.5 mg/kg), eggplant (5.62 mg/kg), round gourds (61.53 mg/kg) [18], leafy brassica [19], passionfruit [4], wolfberry [20], tomatoes and lettuce [21], but also in drinking water [22]. To the best of our knowledge, there are no experimental studies on humans regarding the toxicity of -difenoconazole and/or of its stereoisomers. Still, there are several studies regarding the toxicity of this fungicide on laboratory animals and the environment. Animal studies on the toxic effects of difenoconazole showed slightly acute toxicity when ingested and low acute toxicity produced by dermal and inhalation exposure, but at high doses, this fungicide produced liver tumors [23], developmental toxicity [24] and significant alterations in the different biochemical parameters [25]. Studies regarding the toxic effects of difenoconazole on fish showed that the exposure could produce cardiotoxicity, growth inhibition, developmental abnormalities and endocrine disrupting effects [26,27,28,29].

Regarding the toxicological effects of the stereoisomers of triazole fungicides, literature data showed that both their antifungal activity and the environmental toxicity were stereoselective [30,31,32,33,34], but there has not been focused research on their potential risks to human health. The last edition of the European Food Safety Authority (EFSA) report regarding the possible human toxicity of DFC did not consider the toxic properties of its isomers [19]. Difenoconazole has two chiral centers and, consequently, four stereoisomers: (2*R*,4*R*)-, (2*R*,4*S*)-, (2*S*,4*R*)- and (2*S*,4*S*)-difenoconazole. The marketed product usually contains a ratio of about 60:40 cis:trans diastereomers, with a ratio of 1:1 between cis and trans racemates, meaning 30% of (2*S*,4*R*)-, 30% of (2*R*,4*S*)-, 20% of (2*R*,4*R*)- and 20% of (2*S*,4*S*)-difenoconazole [35]. Due to their distinct molecular configurations, they may interact differently with molecular targets, but little is known regarding the bioactivity and toxicity (both for humans and the environment) of every isomer. The literature data contain only studies concerning the distinct bioactivity and the environmental toxicity of the difenoconazole stereoisomers. It was shown that these stereoisomers had distinct activity against fungi and the degradation rates of the stereoisomers in vegetables and soil were different; the (2*S*,4*S*)-isomer showed the highest toxicity and lowest bioactivity [36]. A study made using mice as model organisms showed that (2*R*,4*R*)-DFC disrupted hepatic lipid metabolism and (2*S*,4*R*)-, (2*R*,4*R*)-, and (2*R*,4*S*)-difenoconazole affected intestinal permeability and gut microbiota [34]. 

As the literature data informs us about several toxicological effects of difenoconazole on laboratory animals and the environment, and that the bioactivity and toxicological effects of difenoconazole were stereoselective, this study addresses possible human health risks resulting from exposure to this fungicide via food and drinking water, and the stereoselectivity of the toxicological effects on humans. To obtain this information, a computational approach was considered. In the last years, in silico methods have been used to assess the ADMET profiles and to predict the toxicological endpoints of numerous drugs and drug-related chemicals [37,38,39,40,41,42,43], but also of other xenobiotics, such as phthalates [44], pesticides [12], artificial sweeteners [45], and industrial chemicals [46]. It emphasizes the applicability of these methods for assessing the biological activity of chemicals.

## 2. Results and Discussions

### 2.1. ADMET Profiles of the Stereoisomers of Difenoconazole

The predictions obtained using ADMETlab 2.0 online tool [47,48] regarding the medicinal chemistry of the stereoisomers of difenoconazole showed that these compounds fulfilled the Lipinski Rule of Five [49] and had good bioavailability. Still, they did not pass the Pfizer [50] and GSK [51] rules and, consequently, were considered potentially toxic compounds (Table 1). Predictions obtained using both ADMETlab 2.0 and admetSAR 2.0 [52,53] online facilities regarding the absorption, distribution and excretion of the stereoisomers of difenoconazole are shown in Table 2. 

It must be highlighted that the major limiting point of these outcomes is the reliability of the predictions, as computational models have limited domains of applicability (AD, areas of the physicochemical, structural, or biological space where the model is expected to be exploitable and the predictions are assumed to be trustable) [54]. In the case of the admetSAR 2.0 and ADMETLab 2.0 online tools, the AD approaches allow for estimating the prediction accuracy for every compound individually and, consequently, to make discriminating predictions with an accuracy close to that of the experimental measurements used for building the models. In the particular case of difenoconazole and its stereoisomers, the predictions were within the applicability domain.

The data presented in Table 2 showed small differences between the values of probabilities computed using ADMETlab 2.0 for the same biological activity of the stereoisomers. No differences were obtained between the values of the probabilities computed for the stereoisomers when using the admetSAR 2.0 online prediction tool. Nevertheless, these outcomes showed that all the difenoconazole stereoisomers had high intestinal absorption—this prediction correlates well with the fulfilment of Lipinski’s rule. Furthermore, good absorption was underlined by the fact that none of the isomers were considered a substrate or inhibitor of the permeability glycoprotein, a cell membrane protein whose role is to pump foreign substances out of cells.Another computational study showed good oral bioavailability for other triazole fungicides [12]. All the difenoconazole stereoisomers showed high plasma protein binding limiting their partitioning from the blood into the tissues where they might be metabolized, thus extending their half-life. This last prediction was in good agreement with the values of clearance, indicating a moderate clearance for every stereoisomer, the highest value for the clearance being registered for (2*S*,4*S*)-difenoconazole. The outcomes of the ADMETlab 2.0 online tool did not predict that the stereoisomers of difenoconazole could penetrate the blood–brain barrier; however, the admetSAR 2.0 online tool predicted a high probability for the permeation of all the stereoisomers of difenoconazole across the blood–brain barrier. Due to this mismatch, another online prediction tool, SwissADME [55], has been considered and its outcome also emphasized the ability of the difenoconazole stereoisomers to penetrate the blood–brain barrier. Difenoconazole is a small (MW = 406 g/mol) and hydrophobic molecule (logP = 4.36), and, taking into account these properties, it is expected to cross the blood–brain barrier by transmembrane diffusion [56].

**Figure 1:**
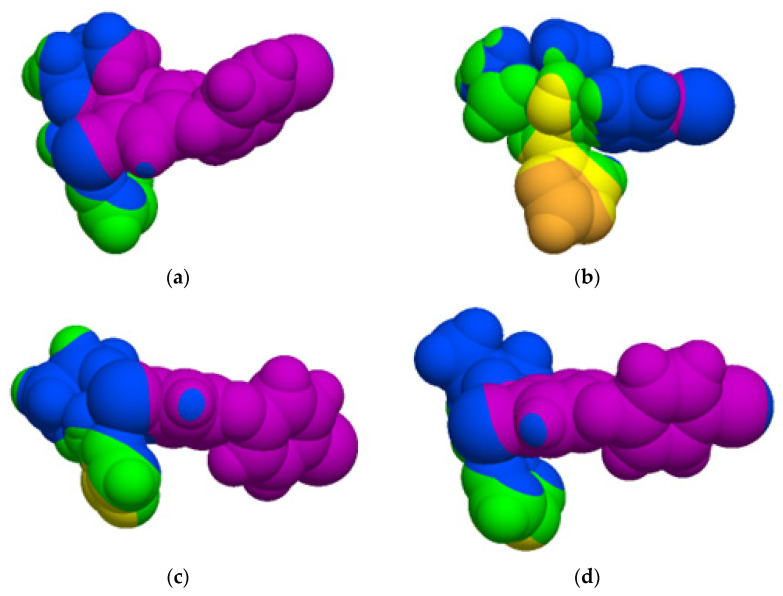
Visualization of the molecular lipophilicity potential (MLP) on the molecular surface of every of the stereoisomers of difenoconazole. The hydrophobic regions are coloured in violet and blue: (**a**) (2*R*,4*R*)-difenoconazole; (**b**) (2*R*,4*S*)-difenoconazole; (**c**) (2*S*,4*R*)-difenoconazole; (**d**) (2*S*,4*S*)-difenoconazole.

Furthermore, the molecular lipophilicity potential (MLP), generated using Molinspiration Galaxy 3D Structure Generator v2021.01 (Slovensky Grob, Slovak Republic, Available online: https://www.molinspiration.com/cgi-bin/galaxy (accessed on 18 July 2022) showed that a larger region of every stereoisomer is highly hydrophobic (blue and violet regions in Figure 1). Consequently, the stereoisomers of difenoconazole are expected to penetrate the blood–brain barrier and potentially produce neurotoxicity. Furthermore, there are published data exemplifying the in vitro neurotoxic effects of propiconazole and tebuconazole, which are other triazole fungicides [57].

The predictions regarding the metabolism of the stereoisomers of difenoconazole are presented in Table 3, and the predictions of their toxicological endpoints are provided in Table 4. 

The data presented in Table 3 show that the probabilities of the interactions of difenoconazole stereoisomers and every of the human cytochromes obtained using the ADMETlab2.0 (Zhejiang, China) online tool were usually not significantly different. There were no registered differences between the values computed using admetSAR 2.0 (Shanghai, China) for the ability of the four DFC stereoisomers to be substrates/inhibitors of cytochromes. The results obtained using ADMETlab 2.0 showed that for all the difenoconazole stereoisomers, there were high values for the probabilities of inhibiting CYP1A2, CYP2C19, CYP2C9 and CYP3A4 and reasonable probabilities from inhibiting CYP2D6. Every one of the stereoisomers showed a high value for the probability of being a substrate for CYP3A4, and (*2S*,*4S*)-difenoconazole exposed a reasonable probability of being a substrate for CYP1A2. The results obtained using the admetSAR 2.0 online tool showed that the probability of difenoconazole stereoisomers being substrates/inhibitors of CYPs were usually lower than those computed using ADMETlab 2.0.

Moreover, there were predictions made by the two online tools that were not in good correlation: (i) ADMETlab 2.0 predicted high values of the probabilities that difenoconazole stereoisomers were inhibitors of CYP1A2, whereas admetSAR 2.0 predicted a low probability of CYP1A2 inhibition; (ii) the predictions made using ADMETlab 2.0 showed that difenoconazole stereoisomers were not considered as substrates for CYP2C9, but those obtained using admetSAR 2.0 were conducive to the high values of the probabilities for these stereoisomers to be substrates for CYP2C9. These contradictory predictions may be due to the distinct models used by the two online tools when predicting the interactions of chemicals with human cytochromes. Impairment of the activity of hepatic cytochromes has been computationally predicted for several triazole fungicides [12]. The interactions of difenoconazole stereoisomers and human CYP are addressed further by using the molecular docking approach and by predicting the metabolism sites.

When using the ADMETlab 2.0 online prediction tool, there were usually small differences between the predicted probabilities for the difenoconazole stereoisomers to conduct to each of the investigated toxicological endpoints, with the probabilities predicted using the admetSAR 2.0 tool being similar for all stereoisomers. Considering the outcomes of the ADMETlab 2.0 prediction tool, all the difenoconazole stereoisomers showed possible induced liver injuries, mutagenicity and skin sensitization potential. The admetSAR 2.0 online tool showed that difenoconazole may produce skin sensitization, carcinogenicity, respiratory toxicity, eye corrosion and irritation. The possibilities to produce cardiotoxicity and skin sensitization have been observed for other triazole fungicides [12].

There were several distinct predictions made by the ADMETlab 2.0 tool regarding the difenoconazole stereoisomers. The (*2S*,*4S*)-difenoconazole exposed reasonable probabilities of producing cardiotoxicity by inhibiting the hERG potassium channel and carcinogenicity. The (*2R*,*4R*)-difenoconazole showed a reasonable probability of producing cardiotoxicity. These results were in agreement with published data obtained through animal studies and highlighted that the fungicide produced liver tumors [23] and developmental toxicity [24], respectively, with the data illustrating that the exposure of fish to difenoconazole had several toxicological effects, including cardiotoxicity, growth inhibition and developmental abnormalities [26,27,28].

Similar values were obtained using the admetSAR 2.0 tool for the probabilities of the difenoconazole stereoisomers to produce various biological activities, and usually, minor differences were observed between the predicted values obtained using the ADMETlab 2.0 online tool for the activities of the stereoisomers of difenoconazole were because the models used in these applications did not consider important features, such as the molecule’s isomeric characteristics. It was not an unexpected result, being known that the QSAR prediction models are not usually suitable to predict stereoisomers, being aimed to predict biological activities using descriptors representing intrinsic properties of chemical structures. The QSAR modeling is continuously evolving, and models include more diverse types of chemical descriptors [58]. The models integrated in the ADMETlab 2.0 tool seem to be more suitable for stereoisomers screening the models incorporated by the admetSAR 2.0 tool. It also underlines the necessity to develop structure-based models that incorporate stereochemically aware descriptors for predicting the activity/toxicity of chemicals.

### 2.2. Endocrine Disrupting Effects on Humans of the Stereoisomers of Difenoconazole

**Figure 2:**
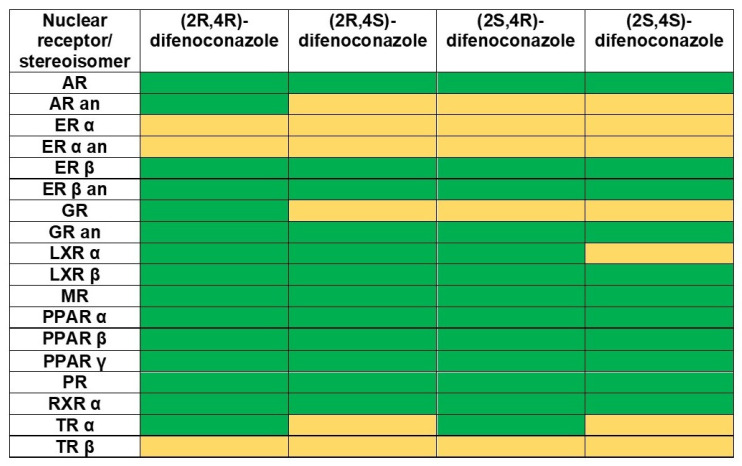
Predictions regarding the endocrine disruption potential of the stereoisomers of difenoconazole: green cells—low probability of binding to nuclear receptors; orange cells—moderate probability of binding to nuclear receptors.

Because there was emphasized that triazole fungicides were able to produce endocrine disruption in mice [11,14] and difenoconazole was able to produce endocrine-disrupting effects in fish [29], the Endocrine Disruptome (Ljubljana, Slovenia) [59] computational tool was also used to predict the possible effects of its stereoisomers on the human endocrine system. The outcomes are shown in Figure 2 and the predictions made by the ADMETlab 2.0 and admetSAR 2.0 online tools regarding the reproductive toxicity and possible damaging effects on the nuclear receptors produced by DFC stereoisomers are presented in Table 5.

Figure 2 emphasizes that the difenoconazole stereoisomers usually showed low probability rates of producing the potential for endocrine disruption. However, all stereoisomers showed a moderate probability of binding to the estrogen receptors α and β and to the thyroid receptor β. Except for (2*R*,4*R*)-difenoconazole, the other stereoisomers exposed moderate probability of binding to the androgen and glucocorticoid receptors. (2*R*,4*S*)- and (2*S*,4*S*)-difenoconazole showed a moderate probability of binding to the thyroid receptor α, and (2*S*,4*S*)-difenoconazole also exposed a moderate probability of binding to the liver X receptor α.

The outcomes obtained using the ADMETlab 2.0 and admetSAR 2.0 tools were not concordant. ADMETlab 2.0 predicted that the difenoconazole stereoisomers did not produce damaging effects on the nuclear receptors, except (2*S*,4*S*)-difenoconazole that showed a medium probability of affecting the estrogen receptor. admetSAR 2.0 showed high probabilities of difenoconazole affecting the estrogen, androgen, glucocorticoid and thyroid receptors and a reasonable probability of affecting PPAR γ. The results obtained using admetSAR 2.0 and Endocrine Disruptome tools were in good agreement with published data revealing endocrine disrupting effects produced by difenoconazole on several species of fishes [29] and the potential of producing endocrine disruption exposed by other triazole fungicides [12]. Furthermore, stereoselective endocrine disrupting effects have been observed for prothioconazole (another triazole fungicide), with S-enantiomer possessing a higher ability to produce hormonal effects via interfering with ERα and TRβ [60].

### 2.3. Assessment of the Interactions of the Stereoisomers of Difenoconazole with Human Cytochromes and Plasma Proteins

**Figure 3:**
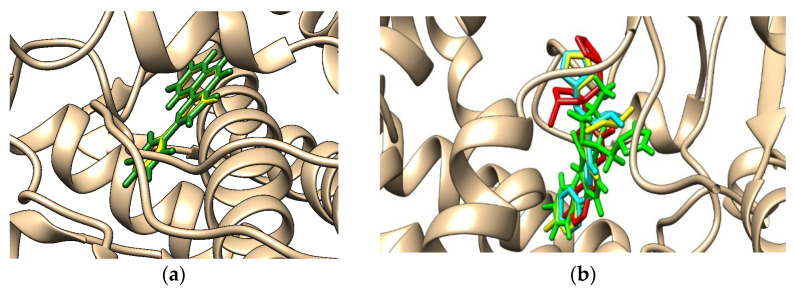
Illustration of the best binding modes of the: (**a**) ligand 2-phenyl-4h-benzo[h]chromen-4-one (forest-green sticks for the docked position and yellow sticks for its position in the crystallographic structure of the complex with CYP1A2); (**b**) stereoisomers (*2R*,*4R*)-difenoconazole (red sticks), (2*R*,4*S*)-difenoconazole (yellow sticks), (2*S*,4*R*)-difenoconazole (green sticks), (2*S*,4*S*)-difenoconazole (cyan sticks) to human cytochrome 1A2 (brown ribbon).

**Figure 4:**
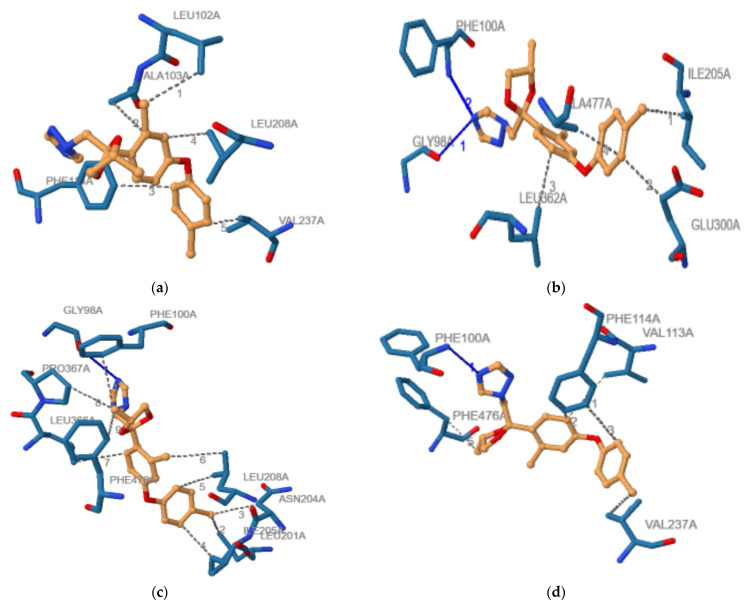
Illustration of the non-covalent interactions between the cytochrome -2C9 and: (**a**) (*2R*,*4R*)-difenoconazole; (**b**) (*2R*,*4S*)-difenoconazole; (**c**) (*2S*,*4R*)-difenoconazole; (**d**) (*2S*,*4S*)-difenoconazole: blue line—hydrogen bond, grey dashed line—hydrophobic interaction.

As some of the outcomes of the ADMETlab 2.0 online tool showed the potential of investigated chemicals to bind to plasma proteins and human cytochromes being mainly involved in the metabolism of xenobiotics, a molecular docking study was also implemented to assess the interactions of the stereoisomers of difenoconazole with these proteins. The result obtained by the molecular docking study and regarding the binding modes corresponding to the highest interaction energies registered for the binding of the ligand 2-phenyl-4h-benzo[h]chromen-4-one (BFH) that is present in the crystallographic structure of the complex with CYP1A2 and respectively of the difenoconazole stereoisomers to the human cytochrome 1A2 are presented in Figure 3. The results regarding the noncovalent interactions between the difenoconazole stereoisomers and CYP2C9, obtained using PLIP online tool [61], are shown in Figure 4.

Figure 3a emphasizes that the docked position of the ligand 2-phenyl-4h-benzo[h]chromen-4-one (BHF, forest green sticks) corresponds to the position of BHF in the crystallographic structure of the complex with CYP1A2 (yellow sticks) and validates the molecular docking outcomes. Figure 3b reveals that (2*R*,4*S*)- and (2*S*,4*S*)-difenoconazole have almost similar binding positions, but there are distinct orientations of the triazole rings of the (2*R*,4*R*)-and especially of (2*S*,4*R*)-difenoconazole. The distinct orientation of the triazole ring of (2*S*,4*R*)-difenoconazole by comparison with the other stereoisomers results in the lower number of hydrogen bonds made by the nitrogen atoms from this ring with the residues of the protein, as is reflected by the spectra of noncovalent interactions (see further, Table 6).

The interacting energies corresponding to the best binding modes of the difenoconazole stereoisomers to the human cytochromes involved in the metabolism of xenobiotic and respectively to the plasma proteins are shown in Table 7. The noncovalent interactions between the difenoconazole stereoisomers and the investigated proteins are shown in Table 7.

For comparison purposes, Table 7 also contains the interacting energies between every protein and its ligand found in the crystallographic structure, and Table 6 contains the noncovalent interactions between the ligands and corresponding proteins: 2-phenyl-4h-benzo[h]chromen-4-one (BFH) for CYP1A2, S-warfarin (SWF) for CYP2C9, 4-hydroxy-3,5-dimethylphenyl)(2-methyl-1-benzofuran-3-yl)methanone (OXV) for CYP2C19, quinine (Q19) for CYP2D6, metyrapone (MYT) for CYP3A4, (2R)-2,3-dihydroxypropyl acetate (JIM) for alpha-1-acid glycoprotein, and diclofenac (DIF) for human serum albumin. The data presented in Table 6 showed that, except for the (*2R*,*4S*)-difenoconazole that did not bind to the catalytic site of CYP2D6, every one of the stereoisomers of difenoconazole was able to bind to the investigated proteins, the interacting energies being usually higher that those corresponding to the best binding modes of the ligands that are present in the crystallographic structures of these proteins. A *t*-test applied with ORIGIN 2021 software (OriginLab. Corp, Northampton, MA, USA) for the data presented in each of the rows from Table 6 and corresponding to the difenoconazole stereoisomers illustrated that, at the 0.05 level, there were significant differences in the interacting energies between each of the stereoisomers of difenoconazole and all of the proteins under consideration.

The noncovalent interactions of difenoconazole stereoisomers with the investigated proteins were mainly hydrophobic (in strong correlation with the hydrophobic character of DFC, logP = 4.36), hydrogen bonds and fewer aromatic donor–acceptor interactions or salt bridges. From one isomer to another, the spectra of the interactions made with the residues of the proteins were quite different, emphasizing the specificity of the binding of each stereoisomer to these proteins and, consequently, the distinct biological activity. It may be noticed that the results obtained using the molecular docking approach were significantly distinct for the stereoisomers of difenoconazole. It underlined that incorporating stereochemically aware descriptors would increase the predictive power of the models used in ADMET screening. Another limitation of the computational methods that are used in ADMET prediction does not consider the concentration of the investigated compound.

The (*2S*,*4S*)-difenoconazole exposed the highest interacting energies with many of the investigated proteins. All the stereoisomers emphasized strong interactions with human HSA and AGP, in good correlation with the predictions of their high plasma protein binding obtained using the admetSAR 2.0 and ADMETlab 2.0 tools. Among CYPs, all the stereoisomers showed strong interactions with CYP3A4 in correlation with the highest values of the probabilities computed using ADMETlab 2.0 for the ability of difenoconazole stereoisomers to act as inhibitors of CYP3A4. The lowest values for the interacting energies have been registered for the interactions of the difenoconazole stereoisomers with CYP2D6, also in correlation with the lowest vales of the probabilities that difenoconazole stereoisomers to be inhibitors of this cytochrome obtained using the admetLab 2.0 online prediction tool. The outcomes of the molecular docking study were in very good agreement with the predicted ADMET profiles of the difenoconazole stereoisomers underlying the reliability of the predictions obtained using the admetSAR 2.0 and ADMETLab 2.0 tools.

### 2.4. Prediction of Metabolism Sites

**Figure 5:**
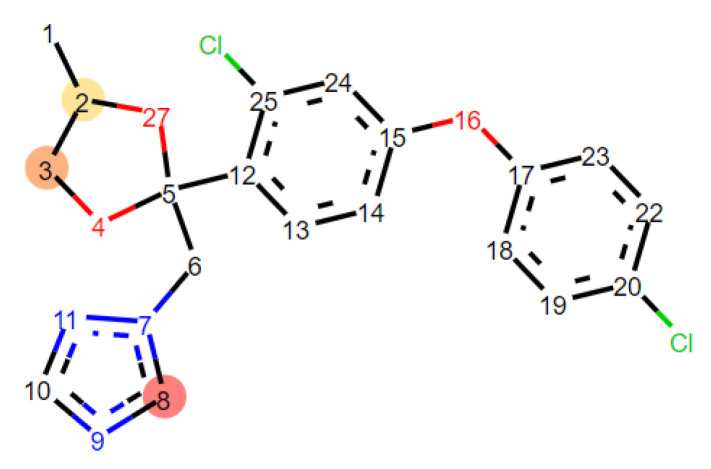
Visualization of the three top-ranking atoms considered as sites of metabolism of the difenoconazole by cytochromes: C8, C3 and C2.

Furthermore, SMARTCyp, a predictive computational tool available online, has been considered to predict the sites of cytochrome P450-mediated metabolism of the stereoisomers of difenoconazole [62]. The outcome of the SMARTCyp online tool is shown in Figure 5 and illustrates that there were at least three carbon atoms predicted as sites of metabolism of difenoconazole by CYP1A2, CYP2C9, CYP2C19, CYp2D6 and CYP3A4. These atoms were ranked by score, with the lowest score revealing the highest probability of being a site of metabolism (Table 8).

The outcome of the SMARTCyp is in good agreement with the results of the molecular docking study. The highest probabilities of the three carbon atoms being sites of CYP metabolism was registered for CYP3A4, in correlation with the highest interacting energies with CYP3A4 registered for all stereoisomers. Similarly, the lowest probabilities of the three carbon atoms being sites of CYP metabolism were registered for CYP2D6, in strong correspondence with the lowest interaction energies registered between the stereoisomers and this cytochrome. These data emphasized that difenoconazole could inactivate the cytochrome P450 enzymes and possibly cause hepatotoxicity as also predicted by ADMET profiles.

## 3. Materials and Methods

### 3.1. Stereoisomers of Difenoconazole

**Figure 6:**
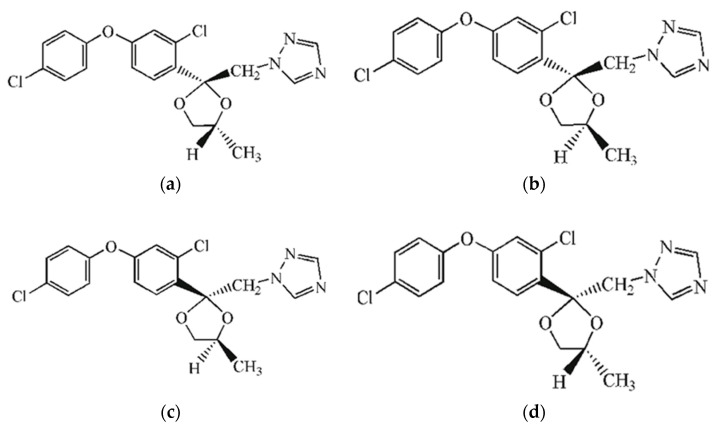
Stereoisomers of difenoconazole (common name, IUPAC name and SMILES formula): (**a**) (*2R*,*4R*)-difenoconazole; (**b**) (*2R*,*4S*)-difenoconazole; (**c**) (*2S*,*4S*)-difenoconazole; (**d**) *(2S*,*4R*)-difenoconazole.

Within this study, we have considered the four stereoisomers of the fungicide difenoconazole, [(2*R*,4*S*)-, (2*R*,4*R*)-, (2*S*,4*R*)-, and (2*S*,4*S*)-isomers], as shown in Figure 6. The PubChem database (Available online: https://pubchem.ncbi.nlm.nih.gov/ (accessed on 12 April 2022) [63] has been used in order to extract the IUPAC (International Union of Pure and Applied Chemistry) name, the SMILES (Simplified Molecular Input Line Entry System) formula, 2D formula and preliminary information regarding the possible toxicity of every isomer.

### 3.2. Prediction of the Human Health Hazards of Difenoconazole

The ADMElab 2.0 (Zhejiang, China, Available online: https://admetmesh.scbdd.com/service/screening/index (accessed on 18 April 2022)) [47,48] and admetSAR 2.0 ((Shanghai, China, Available online: http://lmmd.ecust.edu.cn/admetsar2 (accessed on 25 April 2022) [52,53] tools have been used in order to obtain quantitative predictions regarding the ADMET profiles and toxicological endpoints of the stereoisomers of difenoconazole. The entry data for these tools were the SMILES formulas of the investigated molecules. The outputs consisted of the predictive values for some properties/activities of the investigated compounds and the prediction probabilities for other activities to be manifested by the investigated compounds, respectively. The classification models used by these tools have an accuracy of at least 0.80 and most of the regression models achieved an R^2^ above 0.72 [48,52,53]. Qualitative predictions regarding the ADMET properties have been obtained using SwissADME tool [55]. This tool also uses the SMILES formula of the investigated compound to compute the physicochemical descriptors and the ADMET properties in terms of the presence/absence of biological activity, the accuracy of predictions being between 72% and 94% [55].

Endocrine Disruptome [59] is a computational tool that has been used to predict the possible effects of difenoconazole stereoisomers on the human endocrine system. It considers the molecular docking approach based on the AutoDock Vina algorithm [64] implemented for 14 human nuclear receptors: androgen receptor (AR), estrogen receptors α and β (ER α and ER β), progesterone receptor (PR), glucocorticoid receptor (GR), liver X receptors α and β (LXR α and LXR β), mineralocorticoid receptor (MR), peroxisome proliferator activated receptors α (PPAR α), β/δ (PPAR β), and γ (PPAR γ), retinoid X receptor α (RXR α), and thyroid receptors α (TR α) and β (TR β). The investigated compounds were docked to the structures of these human nuclear receptors, and a docking score of the compound on every receptor structure was computed that was further used to classify the binding of investigated ligand to the nuclear receptors [59].

### 3.3. Molecular Docking Study

This study was performed using the online server SwissDOCK [65] based on the docking software EADock DS [66]. A blind and accurate docking was selected. For comparison and validation purposes, molecular docking was also performed for every investigated protein and its ligand found in the crystallographic structure. The structural files considered for implementing the molecular docking have been selected such as to have a good resolution and to correspond to the complexes between the proteins and inhibitors/substrates. Consequently, the following structural files were extracted from the Protein Data Bank [67]: 2HI4 for CYP1A2, 1OG5 for CYP2C9, 4GQS for CYP2C19, 1W0G for CYP3A4. Similarly, the structural files with the identifiers 3KQ0 and 4Z69 were considered for the α-1-acid glycoprotein (AGP) and for the human serum albumin (HSA), respectively. The preparation of the proteins and ligands for docking and the visualization and analysis of docking results have been performed using Chimera software [68]. The noncovalent interactions between the difenoconazole stereoisomers and the cytochromes and plasma proteins were analyzed using the protein–ligand interaction profiler (PLIP, Dresden, Germany, Available online: https://plip-tool.biotec.tu-dresden.de/plip-web/plip/index (accessed on 8 May 2022) online tool [61].

### 3.4. Prediction of Metabolism Sites

The prediction of metabolism sites was obtained using the SMARTCyp online tool (Copenhagen, Denmark, Available online: https://smartcyp.sund.ku.dk/mol_to_som (accessed on 25 April 2022)) [62]. This tool is based on the 2D structure of the compound and uses Density Functional Theory (DFT) calculations of fragment activation energies to predict which sites in a molecule are most liable to be metabolized by the cytochrome P450 enzymes, which are the most important in the metabolism of xenobiotics. The accuracy of the predictions is at least 76%. [62].

## 4. Conclusions

Several pharmacokinetics and toxicological effects were identified for every one of the stereoisomers of difenconazole: high plasma protein binding correlated with moderate clearance, inhibition of cytochromes, potential to induce liver injuries, neurotoxicity, mutagenic effects and skin sensitization potential. In addition to these hazardous effects manifested by all stereoisomers, the (2*S*,4*S*)-difenoconazole also displayed a reasonable probability of producing cardiotoxicity, carcinogenicity, and negatively affecting numerous nuclear receptors. These effects of the (2*S*,4*S*)-difenoconazole could be enhanced by its moderate clearance. The study's outcome was also in good agreement with literature data emphasizing distinct bioactivity of the difenoconazole stereoisomers and the highest environmental toxicity of the (2*S*,4*S*)-isomer [36].

This limiting point of the outcomes of our study is common to in silico predictions and is represented by the reliability of predictions. The predictions were within the applicability domains of the models, and this increased the credibility of the results. This study also highlighted that molecule’s isomeric structural characteristics should be considered when the models are built to provide readily applicable models.

The potential merit of the outcomes of this study is that it emphasized the possible stereoselective effects of difenoconazole on human health. These predictions can be included in experiments with suitable designs to understand the role of stereoselectivity in human and environmental toxicity of difenoconazole and other chiral pesticides. Taking into account that chiral compounds represent more than 30% of agrochemicals [69], their stereoselectivity should be considered in safety assessments and regulatory decisions.

## Figures and Tables

**Table 1:** Fulfilment of the rules revealing bioavailability (Lipinski) and safety (Pfizer and GSK) of the stereoisomers of difenoconazole.

Stereoisomer/Rule	Lipinski	Pfizer	GSK
(2*R*,4*S*)-difenoconazole	accepted	rejected	rejected
(2*S*,4*R*)-difenoconazole	accepted	rejected	rejected
(2*R*,4*R*)-difenoconazole	accepted	rejected	rejected
(2*S*,4*S*)-difenoconazole	accepted	rejected	rejected

**Table 2:** Predictions regarding the absorption, distribution, and excretion of the stereoisomers of difenoconazole: HIA—human intestinal absorption, P—gp-permeability glycoprotein, BBBP—blood–brain barrier permeation, CL—clearance, HOBA—human oral bioavailability, PPB—plasma protein binding, s—substrate, i—inhibitor.

Stereoisomer/Online Tool/ Activity	ADMETlab 2.0					
	HIA < 30%	P-gp s	P-gp i	BBBP	PPB (%)	CL (mL/min/kg)
(*2R*,*4S*)-difenoconazole	0.003	0.001	0.013	0.021	97.54	11.720
*(2S*,*4R)-*difenoconazole	0.004	0.000	0.005	0.072	97.96	11.662
(*2R*,*4R*)-difenoconazole	0.004	0.001	0.008	0.022	97.69	10.998
(*2S*,*4S*)-difenoconazole	0.003	0.000	0.009	0.064	97.62	12.327
	**admetSAR 2.0**					
	HOBA	P-gp s	P-gp i	BBBP	PPB	
All stereoisomers	0.729	−0.699	−0.660	0.972	1.000	

**Table 3:** Predictions regarding the metabolism of the stereoisomers of difenoconazole: CYP—cytochrome, s—substrate, i—inhibitor.

Stereoisomer/ -Online Tool/ Activity	ADMETlab 2.0									
	CYP1A2		CYP2C19		CYP2C9		CYP2D6		CYP3A4	
	s	i	s	i	s	i	s	i	s	i
(*2R*,*4S*)-difenoconazole	0.493	0.921	0.255	0.922	0.413	0.912	0.268	0.710	0.885	0.945
*(2S*,*4R)-*difenoconazole	0.784	0.941	0.358	0.925	0.155	0.896	0.179	0.775	0.911	0.945
(*2R*,*4R*)-difenoconazole	0.546	0.918	0.294	0.926	0.304	0.923	0.270	0.777	0.911	0.951
(*2S*,*4S*)-difenoconazole	0.735	0.938	0.322	0.924	0.195	0.881	0.164	0.696	0.888	0.934
	**admetSAR 2.0**									
	CYP1A2		CYP2C19		CYP2C9		CYP2D6		CYP3A4	
	i		i		s	i	s	i	s	i
all stereoisomers	0.634		0.792		1.000	0.730	0.895	0.804	0.679	0.864

**Table 4:** Predicted toxicological endpoints of the stereoisomers of difenoconazole: hERG—cardiotoxicity, DILI—drug-induced liver injury, HT—hepatotoxicity, NT—nephrotoxicity.

Stereoisomer/Online Tool/Toxicological Endpoint	ADMETlab 2.0							
	hERG	DILI	AMESToxicity	Skin Sensibilization	Carcinogenicity	Eye Corrosion	Eye Irritation	Respiratory Toxicity
(*2R*,*4S*)-difenoconazole	0.729	0.985	0.960	0.903	0.671	0.003	0.682	0.020
*(2S*,*4R)-*difenoconazole	0.705	0.987	0.926	0.910	0.487	0.003	0.226	0.026
(*2R*,*4R*)-difenoconazole	0.627	0.988	0.957	0.916	0.396	0.003	0.503	0.026
(*2S*,*4S*)-difenoconazole	0.807	0.982	0.944	0.900	0.736	0.003	0.266	0.020
	**admetSAR 2.0**							
	HT	AMES toxicity	Skin sensibilization	Carcinogenicity	Eye corrosion	Eye irritation	Respiratory toxicity	NT
all stereoisomers	0.600	0.600	0.846	0.900	0.986	0.967	0.800	0.620

**Table 5:** Prediction regarding the reproductive toxicity and the damaging effects of the stereoisomers of difenoconazole on the nuclear receptors: AR—androgen receptor, AR-LB—androgen receptor ligand binding domain, ER—estrogen receptor, ER-LB—estrogen receptor ligand binding domain, TYR—thyroid receptor, GR—glucocorticoid receptor, PPAR γ—peroxisome proliferator-activated receptor γ.

Stereoisomer/Nuclear Receptor Inhibition	ADMETlab 2.0					
	AR	AR-LB	ER		ER-LB	PPAR γ
(2*R*,4*S*)-difenoconazole	0.000	0.011	0.146		0.029	0.007
(2*S*,4*R*)-difenoconazole	0.001	0.020	0.265		0.057	0.007
(2*R*,4*R*)-difenoconazole	0.001	0.014	0.251		0.086	0.010
(2*S*,4*S*)-difenoconazole	0.003	0.023	0.557		0.133	0.012
	**admetSAR 2.0**					
All stereoisomers	Reproductive toxicity	AR	ER	TYR	GR	PPAR γ
All stereoisomers	0.878	0.869	0.932	0.806	0.897	0.764

**Table 6:** Noncovalent interactions between stereoisomers of difenoconazole and human cytochromes and plasma proteins, respectively. In parentheses is given the number of the noncovalent interactions when it is higher than 1. For comparative reasons, this table also contains the noncovalent interactions between the investigated proteins and ligands that are present in their crystallographic structures: BFH–2-phenyl-4h-benzo[h]chromen-4-one, OXV–(4-hydroxy-3,5-dimethylphenyl) (2-methyl-1-benzofuran-3-yl)methanone, SWF–S-warfarin, Q19–quinine, MYT–metyrapone, (2R)-2,3-dihydroxypropyl acetate (JIM) for alpha-1-acid glycoprotein, and diclofenac (DIF) for human serum albumin.

Stereoisomer/Ligand	Protein and Types of Interactions		
**CYP1A2**			
	Hydrophobic	Hydrogen bonds	aromatic donor–acceptor interactions
BFH	THR118, PHE125, PHE226, PHE260(2), ALA317, ASP320, THR321, LEU382, ILE386, LEU497, THR498	-	PHE226
(2*R*,4*S*)-difenoconazole	THR124, VAL227, PHE256, ALA317, ASP320, LEU382, ILE386	ARG108, ARG137, ARG456	PHE226
(2*S*,4*R*)-difenoconazole	THR124, PHE226, PHE256, PHE260, ALA317, ASP320, THR321, LEU382, ILE386, LEU497	ILE386	PHE226
(2*R*,4*R*)-difenoconazole	THR124, PHE125 (2), PHE226, ALA317, ASP320, ILE386	ARG108, THR124, ARG137, ARG456	-
(2*S*,4*S*)-difenoconazole	THR124, PHE226 (3), VAL227, ALA317, ASP320, LEU382, ILE386	ARG108, ARG137, ARG456	-
**CYP2C19**			
	Hydrophobic	Hydrogen bonds	aromatic donor–acceptor interactions
OXV	VAL113(2), PHE114, ILE205, VAL208, GLU300(2), THR301, ILE362, LEU366, PHE476(2), ALA477	-	-
(2*R*,4*S*)-difenoconazole	ALA106, VAL113, VAL208, LEU237, ALA297, THR301, LEU366,	LEU201	-
(2*S*,4*R*)-difenoconazole	ILE205, ALA297, THR301, ILE362, LEU366 (2)	ASN204	-
(2*R*,4*R*)-difenoconazole	VAL113, PHE114, VAL208, ALA297, THR301, PHE476 (2)	ASN204	PHE114
(2*S*,4*S*)-difenoconazole	ILE205, LEU237, ALA292, ALA297, THR301, PHE476	-	-
**CYP2C9**			
	Hydrophobic	Hydrogen bonds	aromatic donor–acceptor interactions
SWF	ARG97, PHE100(2), VAL113, LEU208, LEU366(2), PRP367, LEU388, PHE476	PHE100, ALA103, ASN217	PHE114, PHE476
(2*R*,4*S*)-difenoconazole	ILE205, GLU300, LEU362, ALA477	GLY98, PHE100	-
(2*S*,4*R*)-difenoconazole	PHE100, LEU201, ASN204, ILE205, LEU208 (2), LEU366, PRO367, PHE476	GLY98	-
(2*R*,4*R*)-difenoconazole	LEU102, ALA103, PHE114, LEU208, VAL237	-	-
(2*S*,4*S*)-difenoconazole	VAL113, PHE114 (2), VAL237, PHE476	PHE100	-
**CYP2D6**			
	Hydrophobic	Hydrogen bonds	aromatic donor–acceptor interactions
Q19	PHE120(2), LEU121(2), VAL370, VAL374	-	-
(2*R*,4*S*)-difenoconazole	This stereoisomer does not bind to the active site of CYP2D6		
(2*S*,4*R*)-difenoconazole	LEU121, THR309, VAL370, VAL374	PHE120	-
(2*R*,4*R*)-difenoconazole	PHE120, LEU121, GLU216, THR309, VAL370, VAL374 (2)	PHE120	GLU216
(2*S*,4*S*)-difenoconazole	PHE120, ALA305, THR309, VAL370, VAL374	PRO435	-
**CYP3A4**			
	Hydrophobic	Hydrogen bonds	Salt bridges
MYT	ILE118, PHE137, ILE301(2), PHE302, PHE304, ALA305, THR309, LEU373, PHE435, ILE443	SER119, SER437	ARG105 (2), ARG130, ARG375, ARG440
(2*R*,4*S*)-difenoconazole	ALA305, THR309, LEU364, ILE369, PHE435, ILE443	ARG105(2), ARG130, ILE443	-
(2*S*,4*R*)-difenoconazole	ILE301, ALA305, THR309, PHE435	ARG105(2), ARG130, ARG440	-
(2*R*,4*R*)-difenoconazole	ILE301, ALA305, ALA370	ARG105(2), ARG130, ARG440, ILE443	-
(2*S*,4*S*)-difenoconazole	ILE301, ALA305, THR309, LEU364, ILE369, PHE435	ARG105(2), ARG130, ILE443	-
**AGP**			
	Hydrophobic	Hydrogen bonds	aromatic donor–acceptor interactions
JIM	ARG90	ARG90	
(2*R*,4*S*)-difenoconazole	PHE32, TYR37, VAL41, VAL92 (2)	ARG90	TYR37
(2*S*,4*R*)-difenoconazole	TYR27, THR47, VAL92, PHE114 (3)	ARG90	-
(2*R*,4*R*)-difenoconazole	PHE32, TYR37, ILE44, THR47, VAL92 (2), PHE114	ARG90	-
(2*S*,4*S*)-difenoconazole	TYR27, PHE32 (2), TYR37, PHE114 (3)	ARG90	-
**HSA**			
	Hydrophobic	Hydrogen bonds	aromatic donor–acceptor interactions/ salt bridges
DIF	ARG218, LEU219, LEU260	LYS199, ARG218	
(2*R*,4*S*)-difenoconazole	LEU219, PHE223, LEU238, ILE264	ARG257	salt bridges LYS199, HIS242
(2*S*,4*R*)-DFC	ARG218, LEU219, ARG222, LEU238	LYS199 (2)	-
(2*R*,4*R*)-difenoconazole	GLN196, LEU219, PHE223, LEU238, VAL241, ILE290	LYS199	-
(2*S*,4*S*)-difenoconazole	TYR150, LEU219, PHE223, ILE260, ILE290	LYS199	aromatic donor–acceptor interactions ARG257

**Table 7:** Interaction energies (ΔG) between the stereoisomers of difenoconazole and human cytochromes (CYP), human serum albumin (HSA), α-1-acid glycoprotein (AGP). For comparative reasons, this table also contains the interacting energies obtained for the docking of the ligands that are present in the crystallographic structures of the investigated proteins: 2-phenyl-4h-benzo[h]chromen-4-one for CYP1A2, S-warfarin for CYP2C9, 4-hydroxy-3,5-dimethylphenyl)(2-methyl-1-benzofuran-3-yl)methanone for CYP2C19, quinine for CYP2D6, metyrapone for CYP3A4, (2R)-2,3-dihydroxypropyl acetate for alpha-1-acid glycoprotein, and diclofenac for human serum albumin.

Protein/Stereoisomer		ΔG (kcal/mol)			
	Ligand in Crystallographic Structure	(*2R*,*4S*)-Difenoconazole	*(2S*,*4R)-*Difenoconazole	(*2R*,*4R*)-Difenoconazole	(*2S*,*4S*)-Difenoconazole
CYP1A2	−8.62	−9.46	−10.45	−9.69	−9.38
CYP2C19	−7.47	−7.64	−7.77	−7.64	−8.83
CYP2C9	−7.73	−8.19	−7.81	−8.05	−8.36
CYP2D6	−8.71	It does not bind in the active site	−7.56	−7.34	−8.06
CYP3A4	−6.67	−11.33	−12.18	−11.31	−11.20
HSA	−7.37	−9.41	−9.67	−10.50	−10.27
AGP	−6.16	−8.48	−9.15	−8.77	−9.18

**Table 8:** These atoms of difenoconazole considered as the main sites of cytochrome P450-mediated metabolism ranked by score.

**All Stereoisomers**	**Cytochrome**	**Score/Atoms**		
		**C8**	**C3**	**C2**
	CYP1A2, CYP3A4	49.00	53.90	54.90
	CYP2C9, CYP2C19	62.30	72.60	73.50
	CYP2D6	63.10	74.20	75.10

## Data Availability

The data presented in this study are available on request from the corresponding author.

## References

[1] Caglayan C., Taslimi P., Türk C., Gulcin İ., Kandemir F.M., Demir Y., Beydemir Ş. (2020). Inhibition effects of some pesticides and heavy metals on carbonic anhydrase enzyme activity purified from horse mackerel (*Trachurus trachurus*) gill tissues. Environ. Sci. Pollut. Res..

[2] Wang S., Xu Y., Pan C., Jiang S., Liu F. (2007). Application of matrix solid-phase dispersion and liquid chromatography–mass spectrometry to fungicide residue analysis in fruits and vegetables. Anal. Bioanal. Chem..

[3] Khan N., Yaqub G., Hafeez T., Tariq M. (2020). Assessment of health risk due to pesticide residues in fruits, vegetables, soil, and water. J. Chem..

[4] Guo Y. (2021). Determination of fungicide residues. Nat. Food.

[5] de Oliveira Mozzaquatro J., Araújo César I., Barbosa Pinheiro A.E., Caldas E.D. (2022). Pesticide residues analysis in passion fruit and its processed products by LC–MS/MS and GC–MS/MS: Method validation, processing factors and dietary risk assessment. Food Chem..

[6] Lin S., Tang T., Cang T., Yu S., Ying Z., Gu S., Zhang Q. (2020). The distributions of three fungicides in vegetables and their potential health risks in Zhejiang, China: A 3-year study (2015–2017). Environ. Pollut..

[7] Grewal A.S., Singla A., Kamboj P., Dua J.S. (2017). Pesticide residues in food grains, vegetables and fruits: A hazard to human health. J. Med. Chem. Toxicol..

[8] Kim K.H., Kabir E., Jahan S.A. (2017). Exposure to pesticides and the associated human health effects. Sci. Total. Environ..

[9] Horsfield A., Wicks T., Davies K., Wilson D., Paton S. (2010). Effect of fungicide use strategies on the control of early blight (*Alternaria solani*) and potato yield. Australas. Plant Pathol..

[10] Jardim A.N.O., Caldas E.D. (2012). Brazilian monitoring programs for pesticide residues in food-Results from 2001 to 2010. Food Control..

[11] Kjaerstad M.B., Taxvig C., Nellemann C., Vinggaard A.M., Andersen H.R. (2010). Endocrine disrupting effects in vitro of conazole antifungals used as pesticides and pharmaceuticals. Reprod. Toxicol..

[12] Gridan I.M., Ciorsac A.A., Isvoran A. (2019). Prediction of ADME-Tox properties and toxicological endpoints of triazole fungicides used for cereals protection. ADMET DMPK.

[13] Roman D.L., Voiculescu D.I., Filip M., Ostafe V., Isvoran A. (2021). Effects of triazole fungicides on soil microbiota and on the activities of enzymes found in soil: A review. Agriculture.

[14] Taxvig C., Hass U., Axelstad M., Dalgaard M., Boberg J., Andeasen H.R., Vinggaard A.M. (2007). Endocrine-disrupting activities *in vivo* of the fungicides tebuconazole and epoxiconazole. Toxicol. Sci..

[15] Reigart J.R., Roberts J.R. (2013). Recognition and Management of Pesticide Poisoning.

[16] Jallow M.F.A., Awadh D.G., Albaho M.S., Devi V.Y., Ahmad N. (2017). Monitoring of pesticide residues in commonly used fruits and vegetables in Kuwait. Int. J. Environ. Res. Public Health..

[17] Jankuloska V., Karov I., Pavlovska G. (2018). Residue analysis of difenoconazole in apple fruits grown in Republic of Macedonia. J. Agric. Sci. Technol..

[18] Akhtar S., Yaqub G., Hamid A., Afzal Z., Asghar S. (2018). Determination of pesticide residues in selected vegetables and fruits from a local market of Lahore, Pakistan. Curr. World Environ..

[19] Anastassiadou M., Bernasconi G., Brancato A., Carrasco Cabrera L., Ferreira L., Greco L., Jarrah S., Kazocina A., Leuschner R., Oriol Magrans J. (2021). Modification of the existing maximum residue levels fordifenoconazole in leafy brassica. EFSA J..

[20] Chen J.N., Lian Y.J., Zhou Y.R., Wang M.H., Zhang X.Q., Wang J.H., Wu Y.N., Wang M.L. (2019). Determination of 107 pesticide residues in wolfberry with acetate-buffered salt extraction and Sin-QuEChERS nano column purification coupled with Ultra Performance Liquid Chromatography tandem Mass Spectrometry. Molecules.

[21] Elgueta S., Valenzuela M., Fuentes M., Meza P., Manzur J.P., Liu S., Zhao G., Correa A. (2020). Pesticide residues and health risk assessment in tomatoes and lettuces from farms of metropolitan region Chile. Molecules.

[22] EPA-HQ-OPP-2020-0082-0004 Difenoconazole. Human Health Risk Assessment for the Establishment of Tolerances with No U.S. Registrations in/on Japanese Persimmon, Olive, and Black Pepper. https://www.regulations.gov/document/.

[23] Zhuang S.L., Bao L.L., Wang H.F., Zhang M., Yang C., Zhou X.Y., Wu Y., Rehman K., Naranmandura H. (2015). The involvement of ER-stress and ROS generation in difenoconazole-induced hepatocellular toxicity. Toxicol. Res..

[24] Registration Decision for Difenoconazole. https://www.canada.ca/content/dam/hc-sc/migration/hc-sc/cps-spc/alt_formats/pdf/pubs/pest/_decisions/rd2015-15/rd2015-15-eng.pdf.

[25] Abd-Alrahman S.H., Elhalwagy M.E., Kotb G.A., Farid H., Farag A.A., Draz H.M., Isa A.M., Sabico S. (2014). Exposure to difenoconazole, diclofop-methyl alone and combination alters oxidative stress and biochemical parameters in albino rats. Int. J. Clin. Exp. Med..

[26] Mu X., Pang S., Sun X., Gao J., Chen J., Chen X., Li X., Wang C. (2013). Evaluation of acute and developmental effects of difenoconazole via multiple stage zebrafish assays. Environ. Pollut..

[27] Teng M., Qi S., Zhu W., Wang Y., Wang D., Yang Y., Li H., Li C., Dong K., Wang C. (2017). Sex-specific effects of difenoconazole on the growth hormone endocrine axis in adult zebrafish (*Danio rerio*). Ecotoxicol. Environ Saf..

[28] Fan R., Zhang W., Jia L., Li L., Zhao J., Zhao Z., Peng S., Chen Y., Yuan X. (2021). Combined developmental toxicity of the pesticides difenoconazole and dimethomorph on embryonic zebrafish. Toxins.

[29] Huang T., Zhao Y., He J., Cheng H., Martyniuk C.J. (2022). Endocrine disruption by azole fungicides in fish: A review of the evidence. Sci. Total Environ..

[30] Smith S.W. (2009). Chiral toxicology: It’s the same thing…only different. Toxicol. Sci..

[31] Garrison A.W., Avants J.K., Jones W.J. (2011). Microbial transformation of triadimefon to triadimenol in soils: Selective production rates of triadimenol stereoisomers affect exposure and risk. Environ. Sci. Technol..

[32] Li L., Gao B., Wen Y., Zhang Z., Chen R., He Z., Kaziem A.E., Shi H., Wang M. (2020). Stereoselective bioactivity, toxicity and degradation of the chiral triazole fungicide bitertanol. Pest. Manag. Sci..

[33] Yang X., Gong R., Chu Y., Liu S., Xiang D., Li C. (2022). Mechanistic insights into stereospecific antifungal activity of chiral fungicide prothioconazole against *Fusarium oxysporum* F. sp. *cubense*. Int. J. Mol. Sci..

[34] Zhang H., Yang G., Bao Z., Jin Y., Wang J., Chen J., Qian M. (2022). Stereoselective effects of fungicide difenoconazole and its four stereoisomers on gut barrier, microbiota, and glucolipid metabolism in male mice. Sci. Total Environ..

[35] European Patent Specification, Difenoconazole Stereoisomeric Composition with Reduced Phytotoxicity, EP 2 950 653 B1. https://data.epo.org/publication-server/document?iDocId=5829367&iFormat=0.

[36] Dong F., Li J., Chankvetadze B., Cheng Y., Xu J., Liu X., Li Y., Chen X., Bertucci C., Tedesco D. (2013). Chiral triazole fungicide difenoconazole: Absolute stereochemistry, stereoselective bioactivity, aquatic toxicity, and environmental behavior in vegetables and soil. Environ. Sci. Technol..

[37] Isvoran A., Ciorsac A., Ostafe V. (2017). ADME-Tox profiling of some low molecular weight water soluble chitosan derivatives. ADMET DMPK.

[38] Roman M., Roman D.L., Ostafe V., Ciorsac A., Isvoran A. (2018). Computational assessment of pharmacokinetics and biological effects of some anabolic and androgen steroids. Pharm. Res..

[39] Roman D.L., Roman M., Som C., Schmutz M., Hernandez E., Wick P., Casalini T., Perale G., Ostafe V., Isvoran A. (2019). Computational assessment of the pharmacological profiles of degradation products of chitosan. Front. Bioeng. Biotechnol..

[40] Roman D.L., Isvoran A., Filip M., Ostafe V., Zinn M. (2020). In silico assessment of pharmacological profile of low molecular weight oligo-hydroxyalkanoates. Front. Bioeng. Biotechnol..

[41] Dascalu D., Roman D., Filip M., Ciorsac A., Ostafe V., Isvoran A. (2020). Solubility and ADMET profiles of short oligomers of lactic acid. ADMET DMPK.

[42] Wu F., Zhou Y., Li L., Shen X., Chen G., Wang X., Liang X., Tan M., Huang Z. (2020). Computational approaches in preclinical studies on drug discovery and development. Front. Chem..

[43] Roman D.L., Ostafe V., Isvoran A. (2021). Computational assessment of chito-oligosaccharides interactions with plasma proteins. Mar. Drugs..

[44] Craciun D., Dascalu D., Isvoran A. (2019). Computational assessment of the ADME-Tox profiles and harmful effects of the most common used phthalates on the human health. Studia UBB Chem..

[45] Voiculescu D.I., Ostafe V., Isvoran A. (2021). Computational assessment of the pharmacokinetics and toxicity of the intensive sweeteners. Farmacia.

[46] Alves V.M., Muratov E.N., Zakharov A., Muratov N.N., Andrade C.H., Tropsha A. (2018). Chemical toxicity prediction for major classes of industrial chemicals: Is it possible to develop universal models covering cosmetics, drugs, and pesticides?. Food Chem. Toxicol..

[47] Dong J., Wang N.N., Yao Z.J., Zhang L., Cheng Y., Ouyang D., Lu A.P., Cao D.S. (2018). ADMETlab: A platform for systematic ADMET evaluation based on a comprehensively collected ADMET database. J. Cheminform..

[48] Xiong G., Wu Z., Yi J., Fu L., Yang Z., Hsieh C., Yin M., Zeng X., Wu C., Chen X. (2021). ADMETlab 2.0: An integrated online platform for accurate and comprehensive predictions of ADMET properties. Nucleic Acids Res..

[49] Lipinski C.A., Lombardo F., Dominy B.W., Feeney P.J. (2001). Experimental and computational approaches to estimate solubility and permeability in drug discovery and development settings. Adv. Drug Deliv. Rev..

[50] Hughes J.D., Blagg J., Price D.A., Bailey S., Decrescenzo G.A., Devraj R.V., Ellsworth E., Fobian Y.M., Gibbs M.E., Gilles R.W. (2008). Physiochemical drug properties associated with in vivo toxicological outcomes. Bioorg. Med. Chem. Lett..

[51] Gleeson M.P. (2008). Generation of a set of simple, interpretable ADMET rules of thumb. J. Med. Chem..

[52] Cheng F., Li W., Zhou Y., Shen J., Wu Z., Liu G., Lee P.W., Tang Y. (2012). admetSAR: A comprehensive source and free tool for assessment of chemical ADMET properties. J. Chem. Inf. Model..

[53] Yang H., Lou C., Sun L., Li J., Cai Y., Wang Z., Li W., Liu G., Tang Y. (2019). admetSAR 2.0: Web-service for prediction and optimization of chemical ADMET properties. Bioinformatics.

[54] Gadaleta D., Mangiatordi G.F., Catto M., Carotti A., Nicolotti O. (2016). Applicability Domain for QSAR Models: Where Theory Meets Reality. Int. J. Quant. Struct. Prop. Relatsh..

[55] Daina A., Michielin O., Zoete V. (2017). SwissADME: A free web tool to evaluate pharmacokinetics, drug-likeness and medicinal chemistry friendliness of small molecules. Sci. Rep..

[56] Banks W.A. (2009). Characteristics of compounds that cross the blood-brain barrier. BMC Neurol..

[57] Sanchez C.L., Souders C.L., Pena-Delgado C.J., Nguyen K.T., Kroyter N., El Ahmadie N., Aristizabal-Henao J.J., Bowden J.A., Martyniuk C.J. (2020). Neurotoxicity assessment of triazole fungicides on mitochondrial oxidative respiration and lipids in differentiated human SH-SY5Y neuroblastoma cells. Neurotoxicology.

[58] Muratov E.N., Bajorath J., Sheridan R.P., Tetko I.V., Filimonov D., Poroikov V., Oprea T.I., Baskin I.I., Varnek A., Roitberg A. (2020). QSAR without borders. Chem. Soc. Rev..

[59] Kolšek K., Mavri J., Sollner Dolenc M., Gobec S., Turk S. (2014). Endocrine Disruptome—An open source prediction tool for assessing endocrine disruption potential through nuclear receptor binding. J. Chem. Inf. Model..

[60] Zhang Z., Du G., Gao B., Hu K., Kaziem A.E., Li L., He Z., Shi H., Wang M. (2019). Stereoselective endocrine-disrupting effects of the chiral triazole fungicide prothioconazole and its chiral metabolite. Environ. Pollut..

[61] Adasme M.F., Linnemann K.L., Bolz S.N., Kaiser F., Salentin S., Haupt V.J., Schroeder M. (2021). PLIP 2021: Expanding the scope of the protein–ligand interaction profiler to DNA and RNA. Nucleic Acids Res..

[62] Rydberg P., Gloriam D.E., Olsen L. (2010). The SMARTCyp cytochrome P450 metabolism prediction server. Bioinformatics.

[63] Kim S., Chen J., Cheng T., Gindulyte A., He J., He S., Li Q., Shoemaker B.A., Thiessen P.A., Yu B. (2019). PubChem in 2021: New data content and improved web interfaces. Nucleic Acids Res..

[64] Trott O., Olson A.J. (2010). AutoDock Vina: Improving the speed and accuracy of docking with a new scoring function, efficient optimization, and multithreading. J. Comput. Chem..

[65] Grosdidier A., Zoete V., Michielin O. (2011). SwissDock, a protein-small molecule docking web service based on EADock DSS. Nucleic Acids Res..

[66] Grosdidier A., Zoete V., Michielin O. (2011). Fast docking using the CHARMM force field with EADock DSS. J. Comput. Chem..

[67] Berman H.M., Westbrook J., Feng Z., Gilliland G., Bhat T.N., Weissig H., Shindyalov I.N., Bourne P.E. (2000). The Protein Data Bank. Nucleic Acids Res..

[68] Pettersen E.F., Goddard T.D., Huang C.C., Couch G.S., Greenblatt D.M., Meng E.C., Ferrin T.E. (2004). UCSF Chimera—A visualization system for exploratory research and analysis. J. Comput. Chem..

[69] Basheer A.A. (2018). Chemical chiral pollution: Impact on the society and science and need of the regulations in the 21st century. Chirality.

